# A Profilometric Study to Assess the Role of Toothbrush and Toothpaste in Abrasion Process

**Published:** 2015-09

**Authors:** Sandeep Kumar, Siddharth Kumar Singh, Anjali Gupta, Sayak Roy, Mohit Sareen, Sarang Khajuria

**Affiliations:** a Dept. of Public Health Dentistry, Sri Aurobindo College of Dentistry, Indore, Madhya Pradesh, India.; b Dept. of Oral Medicine and Radiology, Sri Aurobindo College of Dentistry, Indore, India.; c Dept. of Oral Medicine and Radiology, Consultant, Dafodyl dental Clinic, Kolkata, India.; d Dept. of Oral Medicine and Radiology, Rajasthan Dental College, Jaipur, India.; e Dept. of Oral Medicine and Radiology, Pacific Dental College, Jaipur, India.

**Keywords:** Toothbrush, Tooth-paste, Abrasion, Profilometer

## Abstract

**Statement of the Problem:**

Despite of many studies conducted on toothbrushes and toothpaste to find out the culprit for abrasion, there is no clear cut evidence to pin point the real cause for abrasion.

**Purpose:**

An *in vitro* assessment of the role of different types of toothbrushes (soft/ medium/hard) in abrasion process when used in conjunction with and without a dentifrice.

**Materials and Method:**

Forty five freshly extracted, sound, human incisor teeth were collected for this study. Enamel specimens of approximately 9 mm^2 ^were prepared by gross trimming of extracted teeth using a lathe machine (Baldor 340 Dental lathe; Ohio, USA). They were mounted on separate acrylic bases. The specimens were divided into three groups, each group containing 15 mounted specimens. Group 1 specimens were brushed with soft toothbrush; Group 2 brushed with medium toothbrush and Group 3 with hard toothbrush. Initially, all the mounted specimens in each group were brushed using dentifrice and then the same procedure was repeated with water as control. Profilometric readings were recorded pre and post to tooth brushing and the differences in readings served as proxy measure to assess surface abrasion. These values were then compared to each other. Kruskal Wallis and Mann-Whitney U test were performed.

**Results:**

The results showed that brushing, with water alone, caused less abrasion than when toothpaste was added (*p*< 0.008). When brushed with water, the harder toothbrush caused more abrasion (higher Ra-value), but when toothpaste was added, the softer toothbrush caused more abrasion (*p*< 0.001).

**Conclusion:**

Besides supporting the fact that toothpaste is needed to create a significant abrasion, this study also showed that a softer toothbrush can cause more abrasion than harder ones. The flexibility of bristles is only secondary to abrasion process and abrasivity of dentifrice has an important role in abrasion process.

## Introduction

Effective plaque control is critical to the maintenance of oral health, because dental plaque is the primary etiological factor in the introduction and development of both caries and periodontal disease.(1) Plaque removal with a manual toothbrush represents the most frequently used method of oral hygiene in Western societies. A toothbrush should be able to reach and clean efficiently most areas of the mouth.(2)

The toothbrush is the principal instrument in general use for accomplishing plaque removal as a necessary part of disease control.(3) Many different designs of toothbrushes and supplementary devices have been manufactured and promoted. Depending on the diameter of the bristles, toothbrushes have been categorized as soft (0.2 mm), medium (0.3 mm) and hard (0.4 mm).(4)

Dentifrices have been used in conjunction with toothbrushes since a long time. The use of toothbrush with dentifrice improves the mechanical control of dental plaque.(5) Various studies have found that some degree of abrasivity is needed in toothpaste if satisfactory cleaning of the teeth is to be achieved.(6-7) On the contrary, some studies have found that toothpaste does not have any contributing effect in the mechanical plaque removal.(8) Besides, regular tooth brushing with dentifrices has been considered an etiological factor in gingival recession and tooth wear as reported by various studies.(9-10) The bucco-cervical regions of the teeth are the most vulnerable and the hard tissues mainly affected are cementum and dentin. The consequent lesions are called dental or cervical abrasion.(11)

Besides cleaning of teeth, the injudicious use of toothbrush has been associated with harmful effects on dentition. Some studies have found that hard toothbrushes cause more abrasion than soft brushes.(12-13) On the contrary, some studies have found that soft brushes lead to more abrasion than hard ones.(4, 14) This is explained by the fact that soft bristles have better flexibility and hence, they cover a larger surface area and also retain more toothpaste.

The mechanism is unclear as to how abrasion varies with the use of different types of toothbrushes and the role of toothpaste in abrasion process. Different *in vitro* studies have used profilometer to measure surface abrasivity. Profilometer is a device which can measure changes in surface roughness. It provides roughness average (Ra) values for each profile. The profilometer produces a tracing using digital and analogue hardware and software, and calculates the average surface roughness (Ra) value for the resultant tracing.(15-16) Therefore, this study was undertaken with the objective of *in vitro* assessment of the role of different types of toothbrushes in abrasion process when used in conjunction with and without a dentifrice. To the best of authors’ knowledge, it is the first *in vitro* study done in India to assess the role of toothbrush and toothpaste in abrasion process.

## Materials and Method

This in* vitro *study was conducted in a private dental institution in India. Ethical clearance to conduct the study was taken from the related institution.

**Preparation of acrylic plates with enamel specimens**

Enamel specimens of size 9 mm^2 ^were prepared and then embedded on acrylic bases (Meliodent Cold; Heraeus Kulzer, Hanau, Germany). A total of 45 mounted specimens were prepared. Any stains, food debris, and calculus adhering to the mounted specimens were cleared off. They were further divided into three groups (Group1,2,3). Each group comprised of 15 mounted specimens. All specimens of Group 1 was brushed using soft toothbrush; Group 2 using medium toothbrush and Group 3 using hard toothbrush.

**Construction of customized brushing model**

**Figure 1:**
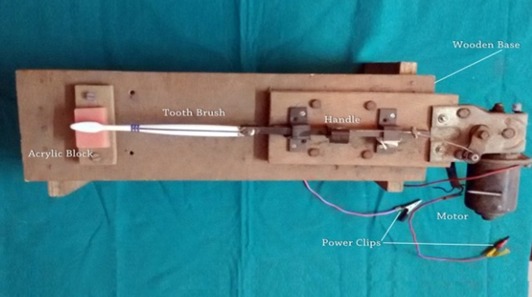
Customized automated brushing machine to deliver uniform force.

In order to deliver uniform force in unidirectional motion, a brushing model was fabricated under expert guidance. The customized brushing model comprised of a motor (Wexco; New Jersey, USA), handle, and a wooden base. This device was electrically operated. The apparatus had a screw and wedge design that facilitated easy replacement of one type of toothbrush with other (Figure 1).

**Selection of toothbrushes**

A soft, medium, and hard toothbrush of the same manufacturer (Kent Refresh; Suffolk, England, UK) was used in the study. All of them had flat trim bristle design. The toothbrushes were firmly fixed over the apparatus using screws. 

**Dontrix gauge**

The tension of the spring was adjusted using Dontrix Gauge (GAC International; Bohemia, New York, USA). The force was maintained at 180±20 grams.(17-18) This force range was selected as it is the normal force which people apply manually during tooth brushing. The Dontrix gauge was used to adjust the tension periodically.

**Dentifrice slurry**

In order to check the role of dentifrice in abrasion process, a standard tooth whitening dentifrice was used in the study (Colgate, INDIA). The toothpaste contained hydrated silica as the main abrasive agent. The other active ingredients were sodium fluoride 0.24% and Triclosan 0.30%. Slurry of the dentifrice was prepared and it was spread over the mounted enamel specimens using a measuring scoop prior to tooth brushing. Each time equal amount of slurry was applied. It was placed over the enamel surface and gently spread over the surface using the toothbrush bristle tips.

**Brushing duration and frequency**

Brushing was carried out for each mounted specimens for duration of 2 minutes, twice a day, for 3 months.

**Profilometer**

**Figure 2:**
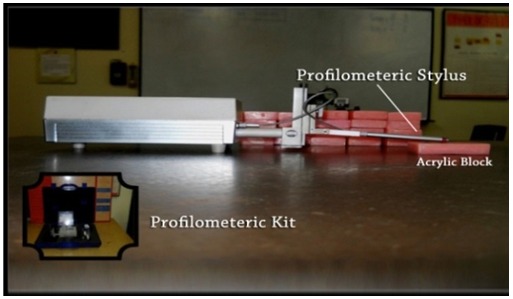
Profilometric kit along with the profilometer

The mean surface loss was evaluated using a profilometer. It provides Ra value (average surface roughness) and difference in Ra value before and after tooth brushing provides proxy measure for assessing surface abrasion. The Ra value for all the 45 mounted enamel specimens were calculated prior and post to tooth brushing and the difference in Ra value (Post-Pre) was used to assess change in surface roughness/ abrasion (Figure 2).

**Test procedure**

The profilometric analysis of all the 45 mounted enamel specimens was carried out prior to tooth brushing. The mean surface roughness value was then calculated for each group. The mounted enamel specimens were then firmly fixed over the wooden base. Initially, soft toothbrush was firmly screwed over the apparatus. The apparatus was so designed that it facilitated only unidirectional movement. No lateral movement was allowed. Slurry of dentifrice was spread over the specimens. The pressure was checked using Dontrix gauge. After this, brushing was carried out for the fixed duration and fixed time period in a direction perpendicular to the long axis of the tooth with a uniform force. The same procedure was repeated using medium and hard toothbrushes. After all the specimens were brushed, all specimens of all the three groups were resent to the lab for profilometric analysis and the Ra values were re-recorded. The differences in profilometric readings (post brushing-pre brushing) were computed and mean values were calculated and compared. This difference in surface roughness change was used as a proxy measure to assess abrasion.

All the 45 mounted enamel specimens were then cleaned thoroughly with water to remove any abrasive particle or stains adhering to the surface. The surface roughness was recorded using the profilometer. The procedure was repeated and all the specimens in all the three groups were subjected to brushing cycle, but this time water was used as a control. No dentifrice slurry was applied. After subjecting all the enamel specimens to brushing regimen, surface roughness was re-evaluated using profilometer. The differences in profilometric analysis were computed and this provided proxy measure of abrasion with water as control.

**Statistical test**

All statistical analyses were performed using SPSS version 20. Kruskall Wallis and Mann-Whitney U test were performed. P value≤0.05 was considered as statistically significant. 

## Results

No significant differences (p value 0.705) were observed in surface roughness between the three groups before tooth brushing. A significant difference (*p*≤ 0.001) was observed between the three groups when the change in surface roughness produced after tooth brushing along with a dentifrice were compared. Post hoc analysis revealed that the change produced in surface roughness was significantly (*p*≤ 0.001) higher in Group 1 when compared to Group 2 and Group 3 (Table 1).

**Table 1:** Mean and standard deviation of surface roughness before and after tooth brushing with a dentifrice between the three groups.

**Groups**	**Number of** **specimens**	**Mean(SD) surface roughness before tooth brushing (Ra1)**	**Mean (SD) surface roughness after tooth brushing with dentifrice (Ra2)**	**Mean (SD) of change in surface roughness** **(Ra2-Ra1)**
Group 1 Soft toothbrush	15	2.91(0.84)	3.51(0.97)	0.60(0.55)
Group 2 Medium toothbrush	15	3.05(0.79)	3.15(0.78)	0.10(0.09)
Group 3 Hard toothbrush	15	3.17(0.73)	3.25(0.75)	0.07(0.04)
p value		0.705	0.364	<0.001*

* p< 0.05: significant difference (Kruskal Wallis test)

No significant differences (*p*= 0.982) were observed in surface roughness between the three groups before tooth brushing. A significant difference (*p*≤ 0.001) was observed between the three groups when the change in surface roughness produced after tooth brushing along with water (control) were compared. Post hoc analysis revealed that the change in surface roughness produced was significantly (*p*≤ 0.001) higher in Group 3 when compared to Group 1 and Group 2 (Table 2).

**Table 2:** Mean and standard deviation of surface roughness before and after tooth brushing with water (control) between the three groups.

**Groups**	**Number of specimens**	**Mean(SD) surface roughness before tooth brushing (Ra1)**	**Mean (SD) surface roughness after tooth brushing with water (Ra2)**	**Mean (SD) of change in surface roughness(Ra2-Ra1)**
Group 1 Soft toothbrush	15	2.96(0.91)	3.00(0.92)	0.04(0.03)
Group 2 Medium toothbrush	15	3.00(0.76)	3.07(0.76)	0.06(0.05)
Group 3 Hard toothbrush	15	2.90(0.83)	3.26(0.76)	0.29(0.23)
p value		0.982	0.544	<0.001*

*  p< 0.05: significant difference (Kruskal Wallis test)

Comparison of surface abrasion produced with and without a dentifrice revealed that a significantly (*p*= 0.008) higher change in surface roughness were observed when the specimens were brushed using a dentifrice (Table 3).

**Table 3:** Comparison of surface abrasion produced with and without a dentifrice

**Categories**	**Number of specimens**	**Mean (SD) of surface roughness before tooth brushing**	**Mean (SD) of surface roughness after tooth brushing**	**Mean( SD) of change in surface roughness**
With dentifrice	45	3.04(0.77)	3.30(0.83)	0.26(0.05)
With water (control)	45	2.97(0.81)	3.11(0.80)	0.13(0.02)
p value		0.569	0.237	0.008*

* p≤0.05: significant difference (Mann Whitney U test)

## Discussion

Various types of toothbrushes available in the market keep the buyer in a state of dilemma as to which one to choose, due to lack of information about the quality of it.(19-20) Moreover, brushing associated with dentifrices continues being the most used and efficient procedure(9) of self-care in the practice of oral hygiene in most countries. However, besides having potential benefits of dental plaque biofilm removal and improving oral health, the injudicious use of toothpaste and toothbrush in causing injuries to dental hard and soft tissues has also been documented; abrasion being most common amongst them.(21-22) Since the role of different types of toothbrushes and dentifrice is not still clear in abrasion process, this *in vitro* study was undertaken to assess the role of different types of toothbrushes and toothpaste in causing abrasion.

This study differs from other previous studies conducted as it involves the use of mounted enamel specimens and not acrylic blocks which were used in other similar studies done in other parts of the world.(4, 23) Also the brushing regimen was performed using an automated brushing device. This device helped to deliver uniform force. Hence, our study findings will be more accurate and will better reveal the role of different types of toothbrushes and toothpaste in abrasion process.

Various studies have shown that different variables influence toothbrush abrasion. These variables include brushing technique, force of brushing, duration and frequency of brushing, and type of brush, in particular filament stiffness.(24) In the present study the brushing technique, brushing force, duration and frequency of brushing were kept constant by construction of a customized brushing apparatus that helped to deliver uniform force. Various studies have recommended the use of customized brushing apparatus to assess the role of toothbrush and toothpaste in abrasion process.(13, 25)

The profilometric readings were recorded by placing the profilometric stylus at the center of each mounted enamel specimens before and after tooth brushing. One of the interesting finding of the study was that when the mounted enamel specimens were brushed for two minutes, twice daily, for 3 months using a customized brushing apparatus, the mean surface loss was seen to be significantly higher in Group 1 (soft toothbrushes) when compared to Group 2 (medium) and Group 3 (hard toothbrushes). Similar findings were reported by FV Teche *et al.*,(23) Dyer D *et al.*,(4) This is explained by the fact that the bristles of soft toothbrushes have more flexibility and hence, the area of bristle contact with the brushed surface is more leading to increased surface loss. Moreover, by reason of greater flexion of bristles, the softer toothbrushes retain more amount of toothpaste which consequently would lead to greater surface loss because of its abrasive contents.

In the present study, it was also found that when the specimens were brushed for 3 months using water as a control, the mean surface loss produced was significantly higher in Group 3 (harder toothbrushes). Also it was observed that brushing with water caused very little abrasion on mounted enamel specimens when compared to the group which was brushed using dentifrices. Similar findings were reported by Tellefsen G *et al.*(14) The authors are of the opinion that the abrasive content of the dentifrice may lead to more surface loss.

Unlike other studies conducted by Dyer *et al.*,(4) FV Teche *et al.*,(23) in which dental acrylic were used to assess abrasion, the present study had the advantage of using mounted enamel specimens with buccal surface brushed with automated brushing machine. It is believed that there are differences in wear of enamel and dentin and enamel being the first layer of tooth are exposed first to tooth brushing. Thus, enamel needs to be protected. Therefore, the study findings will better demonstrate the role of toothbrush and toothpaste in abrasion process. The study used an especially constructed automated brushing apparatus and every care was taken that the tension was adjusted periodically so that the machine delivered uniform force.

However, this *in vitro* study had certain limitations. One of the factors that could be of much importance in methodological resemblance of the dental abrasion *in vitro* researches to its really occurring situation inside the mouth is the simulation of continuous washing action of the saliva and its re-mineralizing protective effects over the worn surfaces of teeth. Few *in vitro* studies have been conducted assessing the role of saliva in abrasion and it was concluded that the abrasion was significantly lowered if saliva was used as a medium.(26-27) In the present study, the effect of saliva and its role in prevention of abrasion was not taken into consideration. Saliva is essential for a lifelong conservation of the dentition.(28) Previous studies carried out by Kumar *et al.*,(29) Hila Hajizadeh *et al.*,(30) Zuryati *et al.*,(31) Kaur and Nandlal(32) have evaluated abrasion produced on dental materials but these studies also had the limitation that the plausiblerole of saliva in abrasion process was not evaluated. Hence, the authors recommend conducting further *in vitro* studies taking saliva into consideration.

Abrasion is of multifactorial etiology and numerous factors affect the abrasion process.(33) Although the authors have done their best to adjust these factors, there are still probabilities that numerous other factors may directly or indirectly influence abrasion process. This *in vitro *study was performed for a short duration; hence, the role of toothbrush and toothpaste for long term use cannot be documented. Moreover, the study did not take into consideration the abrasive nature of toothpastes. Thus, the authors recommend further studies with varying abrasive nature of dentifrices to assess variation in abrasion process with varying abrasivity of dentifrices. Considering the limitations of *in vitro* studies, further research supported by *in vivo* studies need to be conducted before the results can be generalized.

## Conclusion

Abrasion was found to be more when toothpaste was used in conjunction with soft toothbrush. Soft toothbrushes have bristles with more flexibility and have more contact with tooth surface. Also, they retain more toothpaste which is likely to cause more abrasion. When water was used, harder toothbrushes caused more abrasion. Thus, flexibility of bristles is only secondary to abrasion process and abrasivity of dentifrice has an important role in abrasion. 
